# Squamous cell carcinoma of the breast: a case report

**DOI:** 10.1186/1477-7819-6-135

**Published:** 2008-12-21

**Authors:** Elvira R Flikweert, Mans Hofstee, Mike SL Liem

**Affiliations:** 1 Deventer Hospital, Department of Surgery, Postbus 5001, 7400 GC Deventer, the Netherlands; 2 Deventer Hospital, Department of Pathology, Postbus 5001, 7400 GC Deventer, the Netherlands; 3 University Medical Center Groningen, Department of Surgery, Postbus 30.001, 9700 RB Groningen, the Netherlands

## Abstract

**Background:**

Squamous cells are normally not found inside the breast, so a primary squamous cell carcinoma of the breast is an exceptional phenomenon. There is a possible explanation for these findings.

**Case presentation:**

A 72-year-old woman presented with a breast abnormality suspected for breast carcinoma. After the operation the pathological examination revealed a primary squamous cell carcinoma of the breast.

**Conclusion:**

The presentation of squamous cell carcinoma could be similar to that of an adenocarcinoma. However, a squamous cell carcinoma of the breast could also develop from a complicated breast cyst or abscess. Therefore, pathological examination of these apparent benign abnormalities is mandatory.

## Background

Squamous cell carcinoma is a well known malignancy of the skin and other organs surrounded with squamous cells such as the esophagus and the anus. Squamous cell carcinoma of the breast is very rare. It is important to discriminate this entity from malignancies of the skin of the breast or metastasis of a squamous cell carcinoma somewhere else in the body. In the literature only some small series are reported [1-3]. Reported incidences of primary squamous cell carcinoma of the breast vary between 0,1% to less than 0,04% of all breast carcinomas [1-3]. We report a case of primary squamous cell carcinoma of the breast presenting as a usual breast carcinoma. However, in the literature there are examples of less typical presentations, for example starting as an abscess [3].

## Case presentation

A 72 years old white woman presented at the specialized outpatient clinic for breast diseases in the Deventer Hospital in Deventer, The Netherlands. Two weeks earlier, she had discovered a local swelling in her right breast, located behind the nipple. There was no retracted nipple, nor excretion from the nipple. The skin had been red for a while, but this had disappeared spontaneously. The woman was postmenopausal, had given birth to 4 children to whom she had breastfed two. Her family history is relevant for breast cancer, her daughter had breast cancer when she was thirty-five years old. The patient history mentioned a cholecystectomy, hysterectomy and appendectomy and hypertension and atrial fibrillation. She had used some medication against hypertension, an anticoagulant and a tranquilizer.

**Figure 1:**
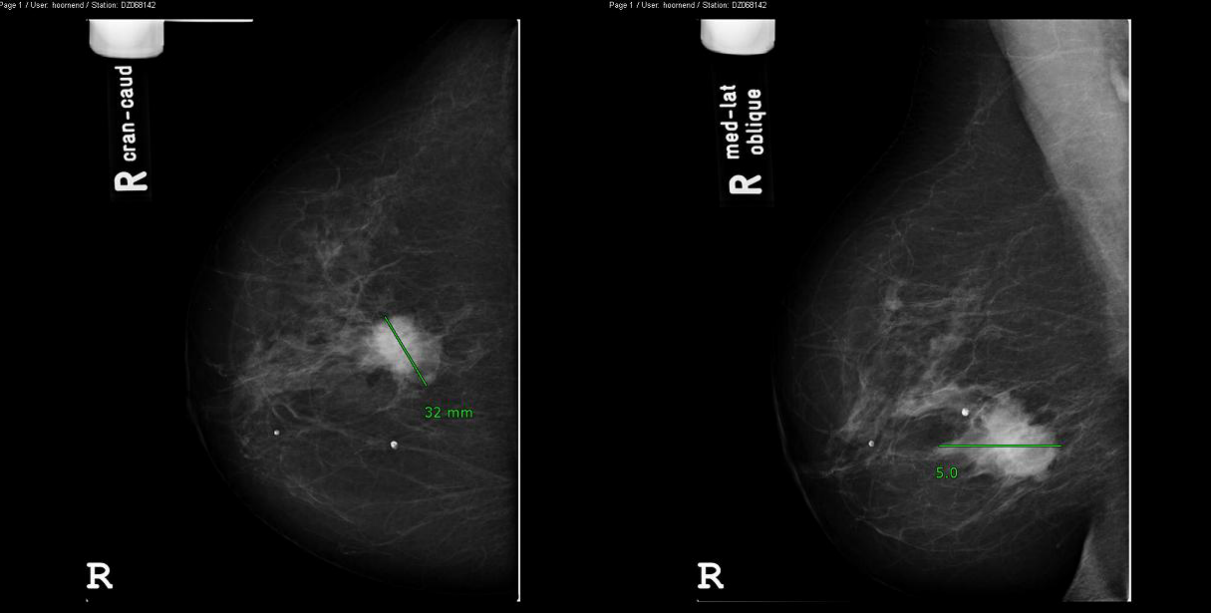
**Mammogram of the of the right breast of the patient in two directions**. Clearly visible the mass, located behind the nipple.

Physical examination revealed an elastic swelling, located centrally in the right breast, measuring about five centimetres across, without fixation to the skin or pectoralis major muscle fascia. The tumour appeared malignant. No abnormalities were observed in the left breast, nor in axillar or supraclavicular lymph nodes. A digital mammogram was performed and showed a mass of 32 millimetres with spiculated margins, positioned five centimetres behind the nipple. Ultrasound of the lesion confirmed this. The radiologist classified the mass as a suspicious abnormality (figure 1). A fine-needle aspiration (FNA) of the swelling was taken. The pathology report confirmed the presence of a malignancy. The pathologist described atypical epithelial cells with polymorphism of the nucleoli, and the conclusion was adenocarcinoma of the breast. An additional ultrasound of the right axilla was performed. It showed a lymph node of 1.5 centimetre, without pathological characteristics. The patient and her family were informed thoroughly about the different treatment possibilities. The decision was made to perform a mastectomy and a sentinel node procedure.

**Figure 2:**
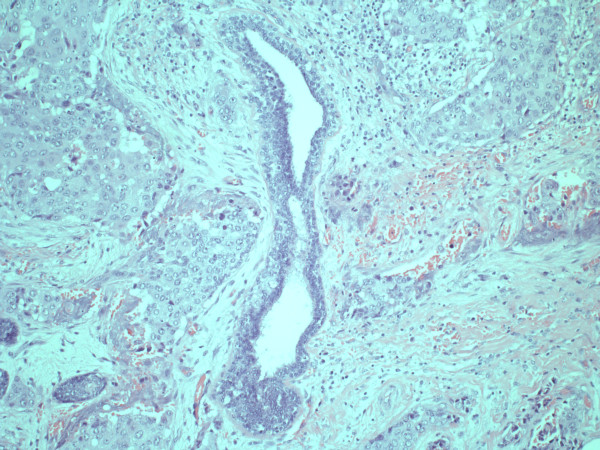
**Squamous cell carcinoma surrounding a pre-existent milk duct, central in the picture**.

Under the nipple, subcutaneously, 44 megabecquerel Technetium^99 ^nanocolloid was injected five hours before the operation. However on the scan made just before the operation, there was no sentinel node visible. At the beginning of the operation patent blue dye was injected to locate the sentinel node. During the operation three blue lymph vessels were seen. There were, however, more enlarged lymph nodes without blue coloring but with pathologic aspect. It was decided to perform a regular lymphadenectomy of the axilla, without removing the highest level nodes. The postoperative course was uncomplicated. She left the hospital five days after the operation, the drains were removed prior to discharge. Pathological examination showed a locally cornified squamous cell carcinoma with a mitosis activity index of more than 20 (figure 2). The conclusion was a radical excision of a moderate differentiated squamous cell carcinoma of the breast, with a size of four centimeters. The Bloom Richardson score was eight, this means high grade malignant. In the preparation eleven lymph nodes were found of which two had metastasis of squamous cell carcinoma. There was no metastasis in the lymph nodes located right underneath the axillary vein. Hence, the tumour was classified as pT2N1Mx breast carcinoma. Determination of the hormone receptors showed positivity for estrogen receptor, the progesterone receptor identification was negative. There was no amplification of the her2neu receptor.

The case was discussed in the multidisciplinary oncology conference. The decision was made to treat this patient according to the Dutch national guidelines for adjuvant treatment with breast carcinoma, just like an adenocarcinoma. Patient was thus started on hormonal therapy: intitially tamoxifen 20 mg daily for two and a half year and an aromatase inhibitor hereafter for the same period. The patient had no other complaints or signs of another tumour.

### Clinical course

A year after the operation there were no indications for relapse nor for metastasis or a skin tumor. The tamoxifen was replaced by an aromatase inhibitor because of side-effects, mostly nausea.

Almost two years after the operation she visited the outpatient medical oncology clinic because of fatigue, anorexia and weight loss. Laboratory investigation showed hypercalcemia. Further evaluation with bone scintigraphy and an ultrasound of the liver showed both, bone and liver metastases. The metastatic disease in the lever was proven by FNA. She was briefly admitted to the clinical ward and was transferred to a hospice facility, where she died shortly afterwards.

## Discussion

Primary squamous cell carcinoma of the breast is very rare. It is called primary pure squamous cell carcinoma when the malignant cells are all of the squamous cell type, there is no relation with the skin and if there is no indication for a primary location somewhere else in the body [4,5]. It is noteworthy to distinguish this type from mixed tumours, where some patches of squamous cells can be found in adenocarcinoma of the breast and from metastasis of squamous cell carcinoma of an origin somewhere else. The etiology and pathogenesis of squamous cell carcinoma of the breast is still unclear. It has been suggested that it may be a very extreme form of squamous cell metaplasia, developing into an adenocarcinoma. This could also explain the mixed forms [6]. Moreover, squamous cell metaplasia is also seen in cysts, chronic inflammations, abscesses and adenofibromas[2]. If these disorders may evolve into carcinomas, this may explain the occurrence of primary squamous cell carcinoma. This hypothesis is further supported by many cases, where primary squamous cell carcinoma is reported after its initial appearance as a benign disorder (abscess or after implantation of a breast prosthesis or after radiation therapy) [2,3,5,7-9]. In our case, however, there was no such pre-existent abnormality. Nonetheless, she did report some inflammation before her presentation at our clinic. In the literature this type of breast carcinoma occurs merely in elderly women. In addition to a presentation with inflammation, the average size of the tumour is larger than adenocarcinoma of the breast [1,2,4]. There are no typical findings on the mammogram. Ultrasound may show a complicated cyst or an inflammatory process. A biopsy should be obtained. In our case, fine-needle aspiration showed malignant cells. The conclusion of the report, adenocarcinoma, was incorrect. In retrospection, it was not justified to draw that conclusion. In one case a correct diagnosis was made on the basis of FNA alone [10].

Squamous cell carcinomas are reported to result in less lymphatic spread than adenocarcinomas. In 10–30% of cases there is lymph node infiltration at the time of surgery [1,2]. In contrast, about 30% of the patients will develop distant metastasis. Squamous cell carcinomas are generally hormone receptor negative [1-5]. It is recommended to give patients similar adjuvant therapy but the radiosensitivity of squamous cell carcinomas is uncertain. The five year survival is 67% in a small retrospective series of eleven patients [2].

Whether investigations, such as PET scans, in search of distant metastases or a primary squamous tumour site should be performed is still a matter of debate [11]. In our patient, further investigation was initially unwarranted because we had no suspicion that the estrogen positive breast tumour was a distant metastasis of an unknown primary squamous site.

## Conclusion

Primary squamous cell carcinoma of the breast is rare. Its existence and possible evolution of an apparently benign disorder underlines the importance of pathological examination of complicated cysts and breast abscesses.

## Consent

Written informed consent was obtained from the patient for publication of this case report and accompanying images. A copy of the written consent is available for review by the Editor-in-Chief of this journal.

## Competing interests

The authors declare that they have no competing interests.

## Authors' contributions

ERF and MSLL initiated and co-wrote the paper, they also took part in the care of the patient. MH examined the specimen and took care of the illustrations.
